# Contradictory Immune Response in Post Liver Transplantation Hepatitis B and C

**DOI:** 10.1155/2014/814760

**Published:** 2014-08-24

**Authors:** Akinobu Takaki, Takahito Yagi, Kazuhide Yamamoto

**Affiliations:** ^1^Department of Gastroenterology and Hepatology, Okayama University Graduate School of Medicine, Dentistry, and Pharmaceutical Sciences, 2-5-1 Shikata-cho, Kita-ku, Okayama 700-8558, Japan; ^2^Department of Gastroenterological Surgery Transplant and Surgical Oncology, Okayama University Graduate School of Medicine, Dentistry, and Pharmaceutical Sciences, 2-5-1 Shikata-cho, Kita-ku, Okayama 700-8558, Japan

## Abstract

Hepatitis B and C often progress to decompensated liver cirrhosis requiring orthotopic liver transplantation (OLT). After OLT, hepatitis B recurrence is clinically controlled with a combination of hepatitis B immunoglobulin (HBIG) and nucleos(t)ide analogues. Another approach is to induce self-producing anti-hepatitis B virus (HBV) antibodies using a HBV envelope antigen vaccine. Patients who had not been HBV carriers such as acutely infected liver failure or who received liver from HBV self-limited donor are good candidate. For chronic HBV carrier patients, a successful response can only be achieved in selected patients such as those treated with experimentally reduced immunosuppression protocols or received an anti-HBV adaptive memory carrying donor liver. Hepatitis C virus (HCV) reinfects transplanted livers at a rate of >90%. HCV reinfected patients show different severities of hepatitis, from mild and slowly progressing to severe and rapidly progressing, possibly resulting from different adaptive immune responses. More than half the patients require interferon treatment, although the success rate is low and carries risks for leukocytopenia and rejection. Managing the immune response has an important role in controlling recurrent hepatitis C. This study aimed to review the adaptive immune response in post-OLT hepatitis B and C.

## 1. Introduction

Hepatitis B virus (HBV) infection and hepatitis C virus (HCV) infection are the main causes of end-stage liver disease requiring orthotopic liver transplantation (OLT). However, the post-OLT course is quite different between the two types of hepatitis. The post-OLT hepatitis B recurrence rate is >80% without any prevention, while >90% of recurrent infections can be controlled with a combination of hepatitis B immunoglobulin (HBIG) and nucleos(t)ide analogues (NAs) [1]. Non-OLT chronic hepatitis B patients are treated with antiviral proliferative NA agents, with >90% long-term control with minute side effects. The first commercially available NA, lamivudine (LAM), produced a rapid and definite short-term antiviral response, but 15–20% of the patients experienced annual recurrence of resistant virus and 70% of them did so after 5 years [2]. Fewer than 3% of patients treated with newer NAs such as entecavir (ETV) or tenofovir (TDF) experience resistant virus; these newer NAs are accepted as first-line and long-term treatment [3, 4].

After OLT, more than half of the patients become reinfected. It is difficult to eradicate the virus once these patients become reinfected [5].

The mechanism for controlling HBV viral recurrence is direct viral replication control by a combination of NAs and HBIG as passive immunoprophylaxis [6]. As HBIG combination therapy has important roles, the B-cell-related adaptive immune response appears to play a role in controlling HBV after OLT. However, as HBV-induced hepatitis is characterized by T-cell immune response, both B- and T-cell adaptive immune responses have vital roles [7]. When active immunization of patients with HBV vaccine is performed, HBV-specific and non-HBV-specific immune responses can be obtained.

HCV reinfects >90% of patients, with more than half of these patients developing chronic hepatitis requiring interferon- (IFN-) based antiviral treatment [8]. Non-OLT chronic hepatitis C patients have been treated with IFN-based immune reaction-related treatment. Recently, pegylated IFN (Peg-IFN) has been used in combination with ribavirin and, more recently, with the addition of a direct-acting antiviral agent (DAA) targeting the HCV nonstructural protein (NS) 3/4A protease [9]. Single Peg-IFN resulted in only 30% of patients experiencing sustained viral response (SVR), representing undetectable HCV-RNA longer than 24 weeks after finishing IFN [10]. This demonstrated >99% viral eradication, while the Peg-IFN and ribavirin combination resulted in 50% SVR [10, 11]. Peg-IFN plus ribavirin and telaprevir or simeprevir resulted in >80% SVR for patients with genotype 1, which is the more difficult-to-treat genotype of HCV [12]. All-oral, IFN-free regimens are expected to become commercially available in the near future [13].

HCV-related liver cirrhosis is a common indication for OLT [14]. However, HCV persists in almost all post-OLT patients. Graft reinfection is universal after OLT [15], leading to high-titer HCV viremia, with cirrhosis developing within 5 years of transplantation in approximately 20% of patients and within 10 years in 50% [16]. Thus, HCV infection after OLT differs completely from chronic hepatitis C (CHC) without transplantation. However, the mechanisms underlying accelerated HCV-induced liver damage after OLT are poorly understood. Several factors appear to be involved in the risk of hepatitis C recurrence, particularly those related to viral and immune responses. Immunosuppressive therapy is a likely cause for the severe accelerated course of HCV-related hepatitis after OLT [16, 17]. In particular, high-dose steroids, immunosuppressive drug combinations, powerful induction treatments, and acute rejection can worsen patient outcomes [18]. The pathology of HCV-related disease reflects immune reactions to virus-infected hepatocytes [7]. In post-OLT settings, immunosuppressive drugs definitely affect the clinical course. The effects of interferon-based treatment are limited to 30–50% of patients, with especially poorer results in post-OLT patients, and also carry the possibility of inducing mortal chronic rejection that should be avoided [19].

In this review, we summarize the aberrant immune system in HBV- and HCV-related hepatitis, together with the changes in these diseases after OLT.

## 2. Immune Mechanisms in Non-OLT HBV- and HCV-Related Hepatitis

### 2.1. Immune Mechanisms in HBV-Related Hepatitis

After infecting a patient once, HBV persists in the liver for the rest of a person's life, even after the patient achieves a clinically cured condition with seroclearance of HBV envelope antigen (hepatitis B surface antigen, HBsAg) and emergence of HBs antibody (HBsAb) [20]. In controlling viral replication, immune function has been revealed to be important, as immunosuppressive treatment for cancer chemotherapy or organ transplantation can induce viral replication even in HBsAg negative with HBsAb positive clinically cured patients and organ transplant recipients [21, 22]. HBV is an enveloped DNA virus containing a relaxed circular DNA genome, which is converted into a covalently closed circular (CCC) DNA that persists in the nucleus of infected cells as minichromosomes [23].

Natural killer (NK) cells work as the innate immune modulator to induce the death of microbial-infected cells with strong cytotoxic activity and the production of high levels of certain cytokines and chemokines in a nonmajor histocompatibility complex- (MHC-) restricted manner distinct from T and B cells [24]. Upon HBV infection, NK cells migrate to the liver, with a decrease in their numbers in the spleen and bone marrow, suggesting the recruitment of NK cells from these organs [24]. As hepatocytes normally express little MHC class I, NK cells may play a more important role in the early defense against HBV infection before the MHC class I expression is upregulated after viral replication in hepatocytes [25].

Antigen-presenting cells (APCs), such as Kupffer cells (liver resident macrophages) and dendritic cells, have important roles in intermediating the innate to adaptive immune responses [26]. Kupffer cells or macrophages behave in both an immunostimulatory and immunoregulatory fashion upon HBV exposure. The addition of HBV particles and HBsAg induces the production of proinflammatory cytokines interleukin- (IL-) 1*β*, IL-6, CXCL-8, and tumor necrosis factor (TNF)-*α* by human CD68^+^ macrophage-enriched cells via NF-*κ*B (nuclear factor kappa-light-chain-enhancer of activated B cells) activation [27]. However, another study reported no such cytokine production with immunoregulatory cytokine transforming growth factor- (TGF-) *β* production [28]. The immune system activates Kupffer cells to eradicate HBV, while HBV evades the Kupffer cell-related pathway to reduce the inflammatory pathway and change the environment to be favorable for survival. Pretreatment of nonparenchymal cells, including Kupffer cells, with HBsAg or HBV virion, abrogates the Toll-like receptor- (TLR-) related antiviral response such as IFN-*β*, interferon-stimulated gene (ISG), or NF-*κ*B. In the liver biopsy specimens of patients with active hepatitis B, Kupffer cells have been revealed to possess higher expression of galectin-9, which is an immunoregulatory molecule [29]. Kupffer cells accumulate around injured hepatic loci and produce several cytotoxic and fibrosis progression-related molecules [30]. However, they also have an important function in scavenging apoptotic hepatocytes, which could function as a bait for inflammation, and depletion of Kupffer cells could induce worsening of hepatitis [31, 32]. Kupffer cells function as both proinflammatory and anti-inflammatory and profibrotic and antifibrotic cells in their environment. Both the hepatitis state and Kupffer cell polarity are needed to understand the immunological pathogenesis in HBV-related hepatitis.

Strong HBV-specific CD8^+^ T-cell responses have been shown to correlate with viral and hepatitis control during acute infection [33]. In chronically infected patients, HBV-specific CD8^+^ T-cell responses are weak and occur with low frequency, while patients with low viral load exhibit multispecific strong responses [34]. The epitopes targeted by the CD8^+^ T cells have been deeply analyzed in HLA-A and -B restricted epitopes, as these have been believed to have antiviral impact [35, 36]. However, a recently characterized HLA-C restricted epitope has also been revealed to have a clinical impact and is an especially frequent allele in patients who live in Southeast Asia [37]. Several factors have been suggested to explain this phenomenon. In HBV-specific CD8^+^ T cells, proapoptotic protein Bcl2-interacting mediator (BIM) is upregulated, naïve T-cell phenotypes such as CD45RA, CD27, CD28, and CCR7 are highly expressed, and immune regulatory molecules such as programmed death-1 (PD-1), cytotoxic lymphocyte antigen 4 (CTLA-4), and T-cell immunoglobulin mucin-3 (TIM-3) are also highly expressed [29, 38–41]. Several experimental trials that were conducted to block such immunoregulatory exhaustion molecules showed reversal of these immunoregulatory conditions [41, 42].

Similar to the CD8^+^ T cells, CD4^+^ T cells have also been found to exhibit a lower response in the acute phase of infection in patients who developed chronic hepatitis later [43]. The CD4^+^ T-cell response in patients who recovered was found to be more frequent, stronger, and more multispecific than that observed later in patients with chronic hepatitis [40]. The IFN-*γ* producing antiviral Th1 response against HBV core has been revealed to be stronger in patients with resolved infection even several years after infection [44].

The humoral immune response has been acknowledged as an avenue for understanding the clinical course of acute and chronic hepatitis B [45]. The antibody responds against viral structural antigens such as the core antigen (HBcAg) and the envelope antigen (HBsAg). Anti-HBcAg IgM antibody (IgM-HBcAb) is accepted as the earliest and most diagnostic marker of acute infection. Anti-HBc IgG antibody (IgG-HBcAb) develops during acute infection and remains positive for the duration of the patient's life [46]. HBsAg emerges in serum from the acute phase of infection and remains when the patient exhibits chronic hepatitis B, while in patients who experience an acute self-limiting course HBsAg could be cleared. Anti-HBs antibody (HBsAb) is a virus-neutralizing antibody recognized as having lower viral and disease activities. The lack of anti-HBs in chronic infection can be attributed to a selective exhaustion of B cells and IL-10 secreting immunoregulatory B-cell expansion [45, 46].

### 2.2. Immune Mechanisms in HCV-Related Hepatitis

**Figure 1:**
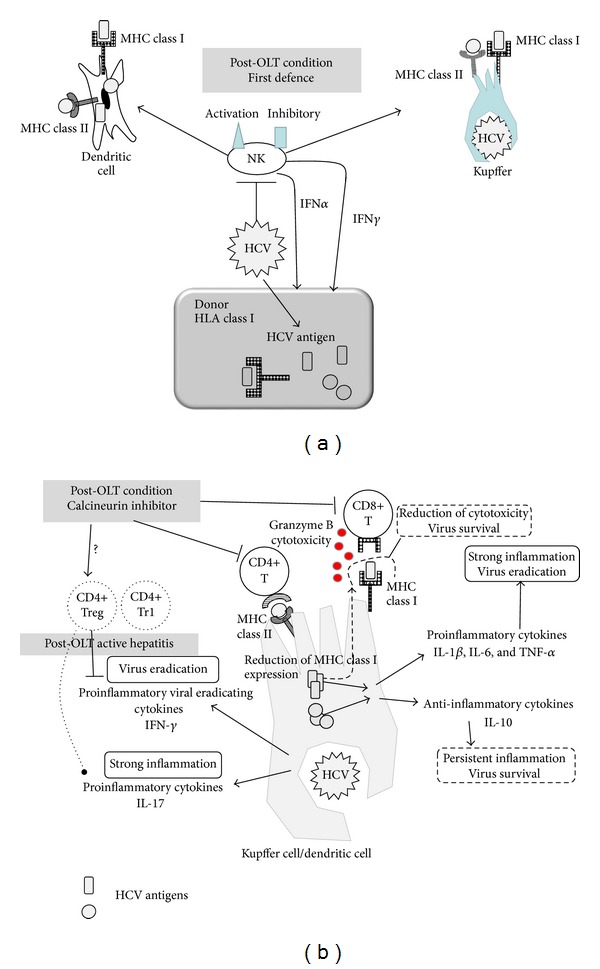
Immune status in chronic hepatitis C and postorthotopic liver transplantation (OLT) hepatitis C. (a) NK cells are the first immunological defense system from hepatitis C virus (HCV). The phenotype defined with the activation or inhibitory receptor gene polymorphism affects the chronic hepatitis C activity and the post-OLT hepatitis activity. The interferon producing function is decreased by HCV proteins. (b) Kupffer cells or dendritic cells (DCs) have important roles in bridging innate and adaptive immune responses. These cells show proinflammatory and anti-inflammatory functions when infected with HCV. These cytokines' balance is well controlled for viral persistence and chronic inflammatory state by viral antigens. After OLT, regulatory T cells might affect to reduce antiviral defence but not to reduce inflammatory cytokines resulting in severer chronic hepatitis. The type 1 regulatory T cells (Tr1) may induce severe hepatitis, while a lower frequency of Tr1 is correlated with hepatitis control with HCV positive status. NK: natural killer cell, OLT: orthotopic liver transplantation, HCV: hepatitis C virus, MHC: major histocompatibility complex, IFN: interferon, Treg: regulatory T cell, and Tr1: type 1 regulatory T cell.

Since HCV is not also a cytopathic virus, immune reactions play a central role in the development of chronic hepatitis (Figure 1) [47, 48]. Innate antiviral responses constitute the first-line defense system against infected virus. HCV disables some innate antiviral systems to escape from the immune pressure [48]. The lack of a strong Th1-type helper T-cell response and cytotoxic T-cell response against HCV leads to chronic infection with this virus [49–51]. High-magnitude, broad, polyfunctional, and sustained T-cell responses correlate with spontaneous recovery [35, 49, 52], but these responses are not correlated with interferon-induced viral clearance [53].

The role of NK cells in chronic hepatitis C is not completely understood. However, as NK cells are the first immunological walls against HCV, much evidence has been uncovered. An NK-cell activating and inhibitory receptor gene polymorphism has been discovered to have roles in the course of HCV infection [54, 55]. As IFN-*α* is the basic treatment for chronic hepatitis C, IFN-producing NK cells have been defined as key immune cells. NK cells can produce IFN-*α*, IFN-*γ*, and TNF-*α* and induce dendritic cell activation and support innate to adaptive immune response bridging [56]. NK cells can also lyse HCV-infected hepatocytes, T cells, and APCs and modulate immune responses [57]. However, HCV itself has been revealed to have a role in the potential inhibition of NK cell function, resulting in chronic hepatitis [58].

Antigen presenting cells such as Kupffer cells, macrophages, or dendritic cells (DCs) behave in both an immunostimulatory and immunoregulatory manner upon HCV exposure, as in HBV [26]. In chronic hepatitis C patients, Kupffer cells are increased and activated as the higher expression markers CD163 and CD33 [59, 60].* In vitro* analysis has revealed that HCV core and NS3-affected Kupffer cells secrete proinflammatory cytokines such as IL-1*β*, IL-6, and TNF-*α* and also immunosuppressive cytokine IL-10 [61]. Proinflammatory cytokine release might explain the induction and persistent inflammation in chronic hepatitis C, while immunosuppressive cytokine release explains the difficulty in the eradication of HCV-infected hepatocytes. The direct effects of HCV on the inflammatory signal in Kupffer cells have been revealed to upregulate the immunoregulatory molecule PD-L1 [62]. Probably, HCV interferes with Kupffer cell-related antiviral activities but induces strong enough inflammatory cytokines to result in chronic inflammation. The effects of Kupffer cells on liver fibrosis progression are similar to those in HBV infection. The Kupffer cells accumulate around inflammatory foci and express cytotoxic molecules such as granzyme B, perforin, and reactive oxygen species to induce inflammation and fibrosis [63].

There are a minimum of two subsets of DCs. Myeloid DCs (mDCs) produce a large amount of IL-12 upon stimulation, while plasmacytoid DCs (pDCs) produce a large amount of IFN-*α* in viral infection [64]. DC function has been reported as broadly impaired in CHC patients [65–68]. However, several reports have indicated contradictory results that DC function is not impaired in CHC patients [69–73]. Most of these reports are studies with mDCs; however, pDCs are also reportedly functionally impaired and reduced by increased apoptosis [74]. Since culture conditions and chronic hepatitis conditions in the patient may change the phenotype of immune cells, functional differences in DCs during chronic HCV infection remain contentious.* In vitro* transfection or the addition of HCV proteins such as core, NS3, or NS4 has been reported to result in reduced function of DCs [75]. Because of the scarcity of* in vitro* culture systems for HCV, these experimental results are also contentious. With the recent establishment of infectious cell culture-produced HCV, impaired pDC functions have been revealed [64].

The role of the humoral immune response in the clearance of HCV is not well understood. After viral clearance, most antibody titers wane despite the persistent T-cell response [35]. A neutralizing antibody response is detectable, even in chronic hepatitis C patients [76]. The target of the response is placed in and around the envelop proteins E1 and E2 and the hypervariable region near the amino terminus of E2 [77]. Neutralization epitopes have been revealed to be masked by extensive glycosylation and by virions covered with lipid droplets and might not be effectively targeted [78, 79]. In addition, as the RNA-dependent RNA polymerase of HCV lacks proofreading activity, it is easy for HCV-RNA to mutate and escape from the host immune pressures [80]. Although 20–30% of infected patients recover from the infection with strong T-cell response memory and possible neutralization antibody B-cell response, they could be reinfected with the virus, indicating difficulties in producing disease-controlling vaccines [81].

Strong HCV-specific CD4^+^ T-cell and CD8^+^ T-cell responses have been shown to be evident in HCV patients with resolved infection, while diminished in patients with chronic hepatitis C [7]. To recognize viral-infected hepatocytes or APCs, viral epitopes should be expressed on the MHC. Interferon upregulates MHC class I expression; however, replicating HCV-RNA reduces that expression [82]. Interferon is released from NK cells and DCs during an early phase of viral infection and has important roles in eradicating HCV, as this is the key drug for treatment [83]. This HCV interference with MHC expression must be one reason why CHC patients show reduced CD8^+^ T-cell responses.

Recent attention has focused on regulatory T cells (Tregs) and their contribution to CHC. Their mechanism of immunosuppression depends on both cell-cell contact and immunosuppressive cytokine secretion [84]. A subpopulation of Tregs that express CD18 and also CD49b-expressing type 1 regulatory T (Tr1) cells have also attracted attention [85], because they produce large amounts of immunosuppressive cytokines such as IL-10 and TGF-*β*, with which they inhibit type 1 and 2 helper responses [86]. Tregs and Tr1 cells may contribute to HCV persistence by suppressing HCV-specific T-cell responses [87–89]. Treg frequencies and activities are apparently higher in CHC patients than in those who have achieved viral clearance [90]. Recently discovered T-cell regulatory molecules such as PD-1, 2B4, and TIM-3 have been revealed to be coexpressed in intrahepatic HCV-specific CD8 T cells, indicating that HCV-induced T-cell functional exhaustion represses viral eradication [91].

Strong innate and adaptive immune responses are responsible for HCV clearance; however, the virus itself affects many sites of the immune system, ameliorating the effective antiviral immune functions. To control NK, Kupffer cells, B cells, or T cells might be difficult as they act in different ways in different CHC conditions.

## 3. Immune Responses in Post-OLT HBV Recurrence Control

### 3.1. Overview of Post-OLT HBV Control with Nucleos(t)ide Analogues and Hepatitis B Immunoglobulin

A multicenter study in Europe in 1993 identified the risk of post-OLT HBV recurrence [92]. The risk was low in patients with acute liver failure who were intolerant of HBV. However, the recurrence rate in patients with liver cirrhosis, especially with high serum HBV-DNA at OLT, was >80% [92]. As the immune system is repressed with steroids and calcineurin inhibitors, recurrent hepatitis B produces severe hepatitis with a high incidence of mortal liver failure. However, present protocols that use NA in combination with long-term HBIG have resulted in >90% control of HBV recurrence [1].

The first trial of long-term HBIG combined with the first-generation NA lamivudine (LAM) was conducted in 1998. Monthly HBIG administration with LAM resulted in all patients surviving for 1 year after OLT without serum HBV-DNA positivity [93]. Subsequent reports also described successful control of HBV recurrence with this combination [94]. The historical progression of controlling post-OLT HBV recurrence is summarized in Table 1. As patients with positive HBV-DNA before OLT were more likely to later have HBV recurrence, to maintain anti-HBs antibody titers >500 IU/L was recommended. If HBV-DNA was negative before OLT, the anti-HBs antibody titer could be reduced to 100–150 IU/L with or without LAM. From the standpoint of cost savings, the HBIG dose requirement was able to be decreased as clinical data accumulated [95–97]. Currently, HBIG is administered as required only when anti-HBs antibody titers fall below target levels. Some reports indicate that only a short duration of HBIG administration is required and that it can be withdrawn several months after OLT [98]. If HBV-DNA was negative at the time of OLT, HBIG could be withdrawn at several months after OLT. For acute liver failure patients who had been infected with the virus shortly before hepatitis development, HBIG could also be withdrawn. Of course, strict monitoring of HBV-DNA and HBV surface antigen (HBsAg) titers should be continued throughout the patient's life.

The mechanism of protection against HBV reactivation by the combination of drugs is not well defined. The cccDNA episome is the transcriptional template for HBV messenger RNA transcripts that encode viral structural and NS proteins and the pregenomic RNA template for reverse transcription and synthesis of the viral genome [5]. NAs inhibit the reverse transcription of pregenomic RNA, resulting in a rapid decrease in serum HBV-DNA, but cannot eliminate the cccDNA reservoir [99]. HBIG contains high-titer antibodies against HBsAg, which is the major component of the envelope of the HBV virion.

The possible mechanisms through which HBIG prevents HBV transmission are that it neutralizes circulating virus by immune complex formation, protects naïve hepatocytes against HBV released from extrahepatic sites through blocking the putative HBV receptor, or anti-HBs antibody internalizes into hepatocytes, interacts with HBsAg, and inhibits HBsAg secretion from cells [100]. To protect against HBV infection of naïve hepatocytes might be difficult, since recent studies have revealed that intrahepatic HBV-DNA is detectable in >50% of even well-controlled patients after OLT [5]. The HBV virion released from the infected cells could be blocked with anti-HBs antibody. In an* in vitro* assay, the internalized antibody was seen to induce the accumulation of intracellular viral particles even after the antibody was removed from the cell culture supernatant [101].

Several new NAs such as adefovir dipivoxil (ADV), entecavir (ETV), telbivudine (LdT), and tenofovir (TDF) have become commercially available [102]. Because of the risk of developing resistance, LAM is no longer recommended as a first-line treatment for hepatitis B. The currently recommended first-line agents are ETV and TDF, which have resulted in a very low emergence of resistance [3, 4]. Such newer NAs are very effective when combined with HBIG even during short duration, post-OLT HBV control [103–109]. Because of low resistance and the powerful antiviral response evoked by ETV and TDF or a combination of NAs, several institutions have developed successful HBIG-free protocols if the HBV-DNA titer is low enough at the time of OLT [103, 110].

As the strong NAs are very effective in HBV control, immune cell-related treatments are not administered, although hepatitis B infection is an immune mediated disease [1].

### 3.2. Adaptive Immune System to Get Anti-HBs Antibody Response with HBV Vaccine

The practice of active immunization of post-OLT recipients with HBV vaccine is emerging. For a successful vaccine response, the immune system has important roles. Most studies report low response rates, even with doubled concentrations or prolonged injections of vaccines (Table 2) [111–115]. Patients who had not been HBV carriers (such as adult patients with acute liver failure due to sexual transmission and nonchronic HBV carriers with anti-HBc antibody-positive donor livers) are good candidates for vaccine administration [112, 116–121]. Patients with acute HBV infection who undergo OLT are often positive for anti-HBs antibody even before OLT and have powerful immune responses. Such patients should respond well to vaccination since they have not developed tolerance to HBV, unlike chronic carriers. However, some HBV carriers have responded to vaccination.

Since noncarriers respond well to HBV vaccination, even under prednisolone and calcineurin inhibitor usage, immune tolerance is expected to play a large role in this process. In non-OLT HBV patients, analysis has revealed that HBsAg-positive newborns had higher regulatory T-cell frequencies and dysfunctional CD8 T cells, which represent immune tolerant status [122]. However, another report analyzing the immunological characteristics of HBsAg-positive young carriers and aged patients with active hepatitis revealed comparable peripheral T-cell proinflammatory cytokine production capacity and HBV-specific IFN-*γ* responses [123]. These findings indicate that tolerant carriers can react with HBV antigens and can show active immunity against HBV vaccination, if regulatory T-cell function diminishes. With good responses to newer NAs after OLT, HBV-DNA decreases even in the liver, and this might recover compressed HBV-specific T cells to react with HBV.

Chronic HBV carrier recipients, including patients with positive HBV-DNA at OLT, do not respond well to HBs-antigen-containing vaccine, with response rates being mostly <30% [114, 115, 119, 124, 125]. Tahara et al. reported 64.7% positive responses to experimentally minimized immunosuppressant treatment [118]. The immune status of these patients was evaluated by a mixed lymphocyte reaction (MLR) assay in response to antidonor and anti-third-party allostimulation using an intracellular carboxyfluorescein diacetate succinimidyl ester- (CFSE-) labeling technique. “Third-party” refers to healthy volunteers with the same blood type as the patients. The autologous lymphocytes, the donor lymphocytes, and the third-party lymphocytes were irradiated and used as the stimulator cells, and the recipients' lymphocytes were used as the responder cells in MLR. The investigators minimized immunosuppression until the donor lymphocytes showed no response as autologous lymphocytes, but third-party lymphocytes showed a positive response. The investigators found that vaccination was successful in patients showing a donor-specific MLR hyporesponse, with a well-maintained response to the third-party stimulus. The vaccine was not successful in patients showing hyporesponse to both the donor and the third party. These results provide encouragement that even immune tolerant liver cirrhosis patients can react with HBV vaccines under lower immunosuppressant protocols after OLT.

Another protocol of repeated vaccine administration resulted in successful immunization in 40% of patients with post-OLT liver cirrhosis [117]. The donors to good responders were the spouses of recipients and had high anti-HBs antibody titers before donation. The spouses with high-titer anti-HBs antibodies were probably infected with HBV by the recipients after marriage, resulting in the anti-HBs antibody boost. The immune systems of these donors should not have developed tolerance to the virus [126]. The adoptive immune transfer of the HBV-specific immune response could be achieved [127].

To successfully transfer immune memory to recipients, the anti-HBs antibody titer of the donors should be high. Luo et al. have shown that a high anti-HBs antibody titer (>1000 IU/L) in donors is essential for adoptive transfer [128]. These results suggest that pre-OLT HBV vaccination for candidate living donors might facilitate improved post-OLT vaccine responses in recipients with liver cirrhosis. Several experimental adjuvant vaccines have also been tried with up to 44.8% success rates [111, 119, 129].

The vaccine response depends on immune tolerance to the virus in both recipients and donors. The liver is the largest immune organ in the abdomen; therefore, it plays a critical role in immune responses. Multiple populations of nonhematopoietic liver cells, including sinusoidal endothelial cells, stellate cells located in the subendothelial space, and liver parenchymal cells, can function as APCs [130]. The viral-specific immune competence of the grafted liver might overcome general immune tolerance to the virus in chronic HBV carriers.

### 3.3. Adaptive Immune System to Get Anti-HBs Antibody Response with HBV Vaccine in HBV Naïve Recipients Who Received Livers from Anti-HBc Antibody Positive Donors

As a shortage of donor organs is a universal problem, anti-HBc positive healthy carriers could be candidate donors. With regard to the above vaccination protocols, non-HBV-related patients who received anti-HBc antibody positive donor livers have fared quite favorably. The post-OLT incidence of* de novo* hepatitis B occurring in anti-HBc antibody-positive donors without prophylaxis is high (33–100%) [22, 131, 132]. These HBV-naïve patients are good candidates for the HBV vaccine because 50–80% tend to respond well [112, 120, 121]. Pre-OLT vaccination is also possible if patients have sufficient time before undergoing OLT. In countries with universal vaccination programs, the recipients might already have anti-HBs antibody and could be boosted with additional vaccination before OLT, resulting in 78% of prospective recipients having a high titer of anti-HBs antibody (>1000 IU/L) [133]. In pediatric patients, the vaccination responses were observed to be good in recipients with higher anti-HBs titers at the time of OLT and lower tacrolimus levels at the time of vaccination [134].

## 4. Adaptive Immune Responses in Post-OLT Hepatitis C Recurrence Control

### 4.1. Overview of Post-OLT Hepatitis C Recurrence and Treatment

As HCV recurrence is observed in almost all the patients who receive OLT, HCV eradication before OLT has been tried, although with unsuccessful outcomes [135, 136].

Post-OLT IFN administration is the only way to achieve better outcomes. HCV genotypes 1b and 4 seem to be negative predictive factors for recurrence because of a lower response to pegylated interferon (Peg-IFN) and ribavirin combination therapy [136]. The host and donor factors associated with poorer outcomes are female gender, older donor age, steatosis of the graft, and the IL-28B single nucleotide polymorphism (SNP) [137–140]. A human genomewide association study recently uncovered many disease-susceptible genes or drug sensitivity-related genes. In CHC patients, the IL-28B gene SNP was found to be related to spontaneous clearance and susceptibility to treatment with Peg-IFN plus ribavirin [141–143]. The combination of recipient and donor IL-28B genetic polymorphism has been revealed to be important in post-OLT HCV treatment outcomes [137].

Recently, direct-acting antivirals such as NS3 protease inhibitors or NS5 polymerase inhibitors or a combination of them have come to represent a new highly effective treatment strategy [144]. The triple combination therapy of Peg-IFN, ribavirin, and a protease inhibitor (telaprevir) has been accepted as a highly effective treatment for non-OLT CHC, producing >75% sustained virological response (SVR) [145]. However, as telaprevir inhibits cytochrome P450 3A4 and reduces the metabolism of calcineurin inhibitors, the trough levels of cyclosporine A (CyA) increase to 4.6-fold and FK 506 (FK) to 70-fold [146]. This phenomenon requires that triple therapy be used with strict care. The second generation protease inhibitor simeprevir very weakly inhibits cytochrome P450 3A4 and is safer than telaprevir. Triple therapy including simeprevir is safer than triple therapy with telaprevir and is currently recommended [147].

### 4.2. Adaptive Immune Responses in Hepatitis C Recurrence

In post-OLT settings, T-cell activities are affected by immunosuppressive therapy [148]. Although the T-cell response is repressed with calcineurin inhibitors, post-OLT CHC patients often show severe hepatitis recurrence with high viral load [14]. In post-OLT CHC patients, the importance of immune reaction has been accepted. Several reports have mentioned that HCV-specific immune responses correlate with post-OLT hepatitis C progression [149, 150]. The frequency of HCV-specific IL-17-secreting CD4^+^ T cells was shown to be increased in severe inflammation in liver fibrosis patients [150]. The serum cytokine profile of these patients with severe recurrence exhibited higher inflammatory cytokines (IL-17, IL-1*β*, IL-6, IL-8, and monocyte chemoattractant protein [MCP]-1), decreased antiviral cytokine IFN-*γ*, and increased IFN-*γ* reducing cytokine IL-10, suggesting the presence of the inflammatory phenotype with repressed antiviral immune response.

Several studies have demonstrated that Tregs induce allograft tolerance [151, 152]. Moreover, Tregs and Tr1 cells are overexpressed in patients with severe hepatitis C recurrence compared with patients with no or minor recurrence [86, 153]. These results suggest that Tregs and Tr1 cells are involved in HCV recurrence after OLT. Because the strength of immunosuppressive therapy and the viral load would be changed after OLT, the time course of the immune response has important roles. Recently, we have shown that Tr1 frequency was repressed in 40 days after OLT under the condition of persistently normal alanine aminotransferase (ALT), even at 3 years after OLT [8]. Tr1, which has a strong IL-10 production capacity, may reduce HCV-specific T-cell responses and induce active hepatitis with ALT elevation. Monitoring Tr1 frequency might be a way to determine which patients would develop active hepatitis. However, HCV-specific CD4^+^ T-cell IFN-*γ* production, which was higher in patients with persistently normal ALT until 3 years after OLT, was found to diminish after 3 years (Tsuzaki R. et al. accepted manuscript for Acta Med Okayama, 2014). This result indicates that, although the adaptive immune response could control hepatitis, the strength of the response might diminish over time. These results from our experience indicate that IFN-based anti-HCV therapy could be applied for patients with higher Tr1 after OLT, who might show active hepatitis until 3 years after OLT. Whether the Tr1 reduction treatment will become the next treatment strategy is not clear, as selective reduction of Tr1 might be difficult. However, as calcineurin inhibitors reduce regulatory T cells, minimum usage of calcineurin inhibitors might be the way this can be accomplished now [154].

### 4.3. Innate Immune Responses in Hepatitis C Recurrence

Innate immune responses have also been identified as HCV targets and could be depressed with respect to their functions. Dendritic cells (DCs) and NK cells are thought to play a central role in the interplay between the innate and adaptive immune responses. Kupffer cells are also involved in post-OLT hepatitis C recurrence, as NF-*κ*B was highly expressed in patients with post-OLT HCV recurrence. However, the specificity for the disease state is not well characterized [155].

In post-OLT settings, blood pDCs decreased after OLT and the pDC product IFN-*α* also decreased. These decreases might affect the recurrence of post-OLT hepatitis C [156].

NK cells have also been deeply investigated with respect to their activities in HCV infection and hepatitis. NK cells are implicated in various viral infections, including HCV and front-line anticancer immune responses. The HCV-E2 protein has been revealed to bind the NK CD81 receptor and decrease the release of IFN-*γ*, resulting in noneffective antiviral responses [157]. Another NK cell receptor, the killer immunoglobulin receptor (KIR), which displays an inhibitory function, has been revealed to be correlated with post-OLT hepatitis C. The KIR-ligand mismatch and recipient KIR2L3 haplotype have been shown to correlate with recurrent hepatitis C [55]. IFN treatment susceptibility of post-OLT HCV recurrence has also been shown to be correlated with the NK receptor haplotype KIR2DS2 [158]. Intravenous administration of living donor perfusate of NK cells could reduce the HCV-RNA increase after OLT [159]. As acutely infected hepatitis C patients show self-recovery at a rate of 20–30%, a strong NK cell response might control hepatitis C even under immunosuppressive treatment.

Adaptive immune response in post-OLT HBV remains a problem that should be investigated, as this virus continues to be difficult to be eradicated from the infected liver. However, the anti-HCV treatment protocol is drastically changing because several clinical trials of new DAA with >80% viral eradication might result in these drugs being introduced to the market; therefore, the importance of investigating the immune system in post-OLT HCV will probably consolidate to selected refractory patients in the next 10 years.

## 5. Conclusion

The adaptive immune response in post-OLT hepatitis B recurrence is hidden under strong antiviral HBIG and NA combination treatment. However, the effectiveness of active immunization is dependent upon adaptive immune responses being effective for patients with non-HBV-related disease who have received anti-HBc antibody-positive donor livers and patients with acute liver failure who are not immune tolerant to HBV. Vaccination is not sufficiently effective for patients with liver cirrhosis; nevertheless, the donor immune memory for HBV and the strength of the immunosuppressant drugs have important roles. Adaptive immune responses, especially of the CD4^+^ and CD8^+^ T cells and the Treg, have strong effects in post-OLT hepatitis C viral recurrence and in recurrent hepatitis activities. The regulatory T cells and Tr1 cells affect the clinical course and could be used as prediction markers. As IFN-based treatments have risks after OLT, forecasting the patient's course with such markers could be beneficial.

## Figures and Tables

**Table 1:** Recent post-OLT HBV prophylaxis with nucleos(t)ide analogue and/or HBIG combination.

	HBV-DNA recurrence (%)	Followup (months)	Reference number	Reported year
Lamivudine + HBIG				
HBIG IV 10000 IU/month	0	13	[93]	1998
HBIG IV to maintain HBsAb >200 IU/L	9.5	21 (2.4–49.1)	[96]	2001
HBIG to maintain HBsAb >70 IU/L	0	16 (9–22)	[97]	2004
HBIG IV to maintain HBsAb >10 IU/L	0	30 (7–73)	[6]	2007
Short course (1 month) HBIG	7	18	[98]	2003
Entecavir + HBIG				
HBIG; IM to maintain HBsAb >100 IU/L	0	41.2 (33–54)	[107]	2012
One year HBIG IM; 2000 IU/month Lamivudine + adefovir or tenofovir, tenofovir, entecavir	0	24 (6–40) post HBIG withdrawal	[106]	2012
One year HBIG IV; dose not specified	3.8	24	[105]	2013
HBIG free with newer nucleos(t)ide analogues regimen				
Lamivudine + adefovir (no HBIG when HBV-DNA below 3log⁡(10)IU/mL)	0	22 (10–58)	[103]	2013
Entecavir, lamivudine + adefovir, tenofovir, entecavir + tenofovir (no HBIG when HBV-DNA below 3.3log⁡(10)IU/mL)	8 (5/6 withdrawn NAs)	21 (1–83)	[110]	2013

OLT; orthotopic liver transplantation, HBV: hepatitis B virus, HBIG; hepatitis B immunoglobulin, HBV-DNA: hepatitis B virus DNA, IV: intravenous administration, IM: intramuscular administration, IU: international unit, HBsAb: anti-hepatitis B s antibody, and NAs: nucleos(t)ide analogues.

**Table 2:** Post-OLT HBV vaccine administration trials.

	Methods	Definition of success	Success rate (%)	Reference number	Reported year
Liver cirrhosis	10–20 *μ*g monthly with experimentally minimized immunosuppressant	HBsAb >100 IU/L without HBIG	64	[118]	2009
	20 *μ*g monthly	HBsAb >100 IU/L 6 months without HBIG	40	[117]	2013
	20 *μ*g monthly	HBsAb >100 IU/L 3 months without HBIG	0	[112]	2011
	40 *μ*g 0, 1, 2, 6, 7, and 8 months	HBsAb >100 IU/L without HBIG	0	[114]	2009
	40 *μ*g 0, 1, and 6 months	HBsAb >10 IU/L without HBIG	82	[113]	2000
	20–40 *μ*g 0, 1, and 6 months	HBsAb >10 IU/L 16 months without HBIG	0	[115]	2010
	20 *μ*g with MPL adjuvant 12 monthly	HBsAb >100 IU/L 18 months without HBIG	44.8	[129]	2010
	Experimental adjuvant vaccine 0, 1, 2, 6, and 12 months	HBsAb >500 IU/L 18 months without HBIG	25	[119]	2005

Acute liver failure	20 *μ*g monthly	HBsAb >100 IU/L 6 months without HBIG	100	[117]	2013
	Experimental adjuvant vaccine 0, 1, 2, 6, and 12 months	HBsAb >500 IU/L 18 months without HBIG	100	[119]	2005
	10–20 *μ*g monthly	HBsAb >100 IU/L without HBIG	66	[118]	2009

Non-HBV-related patients with HBcAb positive donors	20 *μ*g monthly	HBsAb >100 IU/L 3 months without HBIG	83	[112]	2011
	Infant 20–40 *μ*g according to body weight 2–4 times/year	HBsAb >100 IU/L without HBIG	75	[121]	2007
	20 *μ*g 0, 1, and 6 months	HBsAb >100 IU/L 6 months without HBIG	50	[120]	2007

OLT: orthotopic liver transplantation, HBV: hepatitis B virus, HBIG: hepatitis B immunoglobulin, IU: international unit, and HBsAb: anti-hepatitis B s antibody.
